# 609. *In vivo* efficacy of human simulated exposures of cefiderocol (FDC) in combination with ceftazidime/avibactam (CZA) or meropenem (MEM) using in a 72 hour murine thigh infection model

**DOI:** 10.1093/ofid/ofac492.661

**Published:** 2022-12-15

**Authors:** Christian M Gill, Debora Santini, Miki Takemura, Christopher M Longshaw, Yoshinori Yamano, Roger Echols, Roger Echols, Roger Echols, David P Nicolau

**Affiliations:** Hartford Hospital, Hartford, Connecticut; Hartford Hospital, Hartford, Connecticut; Shionogi & Co., Ltd, Toyonaka, Osaka, Japan; Shionogi B.V., London, England, United Kingdom; Shionogi & Co., Ltd., Toyonaka, Osaka, Japan; Infectious Disease Drug Development Consulting, Easton, Connecticut; Infectious Disease Drug Development Consulting, Easton, Connecticut; Infectious Disease Drug Development Consulting, Easton, Connecticut; Hartford Hospital, Hartford, Connecticut

## Abstract

**Background:**

*Acinetobacter baumannii* continues to challenge clinicians as multi-drug resistance limits therapeutic options. CFDC possesses potent *in vitro* and *in vivo* activity however combination therapy has been recommended for *A. baumannii* due to its propensity for multiple resistance mechanisms. The present study utilized clinically relevant exposures of CFDC (2 g IV q8h 3 h infusion) in combination with CZA (2.5 g IV q8h 2 h infusion) or MEM (2 g IV q8h 3h infusion) to evaluate the bactericidal activity and resistance prevention.

**Methods:**

15 clinical *A. baumannii* with the following FDC MICs were assessed: 2 mg/L, n = 3; 8 mg/L, n = 2; ≥ 32 mg/L, n = 10). CZA MICs ranged from 16 - >64 mg/L while MEM MICs ranged from 4 - >64 mg/L. Groups of 6 mice received sham control, CFDC HSR, CFDC + CZA HSR, or CFDC + MEM HSR for 72 h. 1 thigh per mouse was harvested to elucidated bacterial burden at 0 h (baseline) and at 72 h (or when the animal succumbed to infection). Efficacy of the combinations was assessed as change in log_10_ CFU/thigh relative to CFDC HSR. Development of resistance was defined as > 4 fold increase in MIC relative to that from control animals.

**Results:**

Untreated controls resulted in robust growth (3.48±0.67). Against isolates with CFDC MICs of 2 mg/L, 2/3 reached 1-log_10_ kill with CFDC HSR relative to baseline compared with 1/2 and 0/10 isolates with FDC MICs of 8 mg/L and ≥ 32 mg/L, respectively. Against all 15 isolates, CFDC + CZA HSR produced significant kill with a mean -4.77±1.93 reduction in log_10_ CFU/thigh relative to CFDC treated mice (15/15 ≥1-log_10_ kill relative to baseline). Similarly, CFDC + MEM HSR produced a mean reduction of -4.13±2.50 relative to CFDC treated mice (12/15 ≥1-log_10_ kill relative to baseline). Elevated MICs in CFDC treated animals occurred in 3/3 isolates with baseline MICs of 2 mg/L. Of these isolates, 1 developed elevated MICs with CFDC + CZA HSR compared with no isolates with CFDC + MEM HSR.

**Figure 1.**
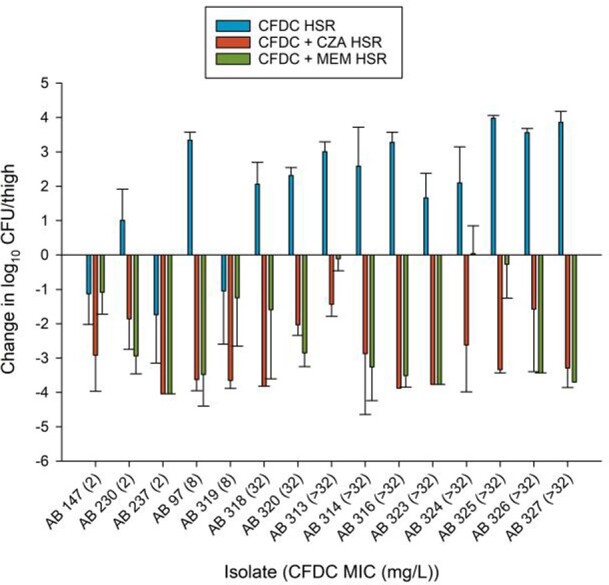

Change in log10 CFU/thigh relative to 0 h control after treatment with cefiderocol HSR, cefiderocol + ceftazidime/avibactam HSR, or cefiderocol + meropenem HSR in the 72 h murine thigh infection model. Cefiderocol MICs are displayed in parentheses.

**Conclusion:**

The present study using clinical exposures of CFDC, CZA, and MEM suggest the enhanced microbiologic activity of these combinations relative to CFDC alone. Combinations also prevented the development of elevated MICs against 2/3 and 3/3 susceptible isolates with CFDC + CZA and CFDC + MEM, respectively. These data support the clinical evaluation of such combinations against *A. baumannii* with high CFDC MICs.

**Disclosures:**

**Christian M. Gill, PharmD**, Shionogi: Grant/Research Support **Miki Takemura, n/a**, Shionogi: Employee **Christopher M. Longshaw, PhD**, Shionogi: Employee **Yoshinori Yamano, PhD**, Shionogi: Employee **Roger Echols, MD**, Shionogi: Advisor/Consultant **Roger Echols, MD**, Shionogi: Advisor/Consultant **Roger Echols, MD**, Shionogi: Advisor/Consultant **David P. Nicolau, PharmD**, Shionogi: Grant/Research Support.

